# Recurrent trauma and non-operative management of femoral fracture in fibrodysplasia ossificans progressiva: a pediatric case report

**DOI:** 10.3389/fped.2026.1777066

**Published:** 2026-10-09

**Authors:** Bicheng Liu, Xiangyu Lv, Yu Wang, Chenglei Zhao, Jingxin Zhao

**Affiliations:** Department of Orthopedics, Affiliated Hospital of Chengde Medical University, Chengde, China

**Keywords:** children, fibrodysplasia ossificans progressive, myositis ossificans progressive, stone man syndrome, trauma

## Abstract

**Background:**

Fibrodysplasia Ossificans Progressiva (FOP) is an ultrarare autosomal dominant disorder caused by ACVR1 gene mutations, leading to dysregulated bone morphogenetic protein (BMP) signaling. It is characterized by congenital great toe malformations and progressive, trauma-induced heterotopic ossification (HO). Misdiagnosis is common, and invasive procedures can exacerbate HO. Current management focuses on flare-up control and trauma prevention.

**Case presentation:**

A 12-year-old Asian boy presented with a left femoral fracture. His history included developmental delay, and HO formation after a prior forearm fracture surgery and other traumas, resulting in progressively limited joint mobility. Physical examination revealed bilateral hallux valgus and multiple hard masses. Imaging confirmed extensive HO. To avoid surgery-induced ossification, closed reduction and casting were performed, alongside comprehensive trauma prophylaxis education and follow-up planning.

**Conclusion:**

This case highlights the critical importance of early recognition of FOP, with particular attention to distinctive great toe abnormalities, to prevent harmful interventions. The established diagnosis and chosen non-operative management underscore the value of a multidisciplinary approach, including genetic testing, conservative orthopedics, and rehabilitation. Enhancing clinician awareness to reduce misdiagnosis and providing timely, evidence-based patient education are essential to mitigate disease progression through rigorous trauma avoidance.

## Introduction

Fibrodysplasia Ossificans Progressiva (FOP) is a rare, highly disabling autosomal dominant disorder with no sex predilection and an estimated global prevalence of approximately 1 in 2 million (1, 2). In 2006, Shore et al. identified the causative factor of FOP. They determined that the genetic locus for FOP resides at 2q23-24 and is caused by mutations in the ACVR1 gene. The ACVR1 gene regulates skeletal and cartilage formation; its mutation leads to aberrant activation of the bone morphogenetic protein (BMP) signaling pathway, consequently triggering progressive and irreversible heterotopic ossification (HO) (3).

The cardinal clinical manifestations of FOP encompass congenital great toe malformations and progressive heterotopic ossification (HO) foci recurrently arising within multiple soft tissues throughout the body, including skeletal muscle, tendons, ligaments, fascia, and aponeuroses. Trauma and invasive clinical procedures significantly accelerate the ossification rate, ultimately leading to joint ankylosis and respiratory impairment in FOP (4–6). Fibrodysplasia ossificans progressiva (FOP) is frequently misdiagnosed as juvenile fibromatosis or sarcoma, potentially leading to unnecessary surgical interventions and clinical procedures that exacerbate disease progression (7, 8). Fibrodysplasia Ossificans Progressiva (FOP) remains incurable, but disease-modifying treatments are now available. Palovarotene, an RARγ agonist approved in several countries, reduces heterotopic ossification by over 50% despite safety concerns such as premature growth plate closure. However, it has not received marketing authorization from the European Medicines Agency (EMA); the EMA's refusal was based on concerns regarding the magnitude of clinical benefit relative to risks, particularly the high incidence of adverse effects (e.g., premature epiphyseal closure, bone growth disturbances) and the limited long-term efficacy data from the Phase III trial. Additional targeted therapies including activin A antibodies and ALK2 inhibitors are under clinical investigation clinical investigation, Whereas high-dose glucocorticoids and NSAIDs remain conventional options for acute flare-ups (7, 9, 10). Due to the exceptional rarity of FOP and the severe consequences of misdiagnosis, detailed documentation of each new case is critically important for clinical practice. This report describes a patient with FOP who experienced multiple traumatic events at different time points, with the aim of contributing to improved diagnostic and therapeutic approaches for future FOP cases.

## Case report

A 12-year-and-1-month-old Asian boy presented to our institution with a left femoral shaft fracture secondary to a fall. His past medical history is significant for FOP-related complications. At age 5, he sustained right ulna/radius fractures after a fall, treated with open reduction and internal fixation at our hospital. Follow-up evaluations later revealed sclerotic masses around the surgical incision (Figure 1), indicative of post-traumatic heterotopic ossification. Prior to this, other traumatic injuries to his lower back and hips, managed with rehabilitation at local hospitals, had resulted in multiple bony masses over the lumbosacral region, accompanied by progressive restriction of flexion-extension in his left knee joint. Throughout these episodes, the patient experienced typical acute flare-ups characterized by local pain, swelling, and warmth, which gradually subsided but left behind hard, ossified masses and functional limitations.

**Figure 1:**
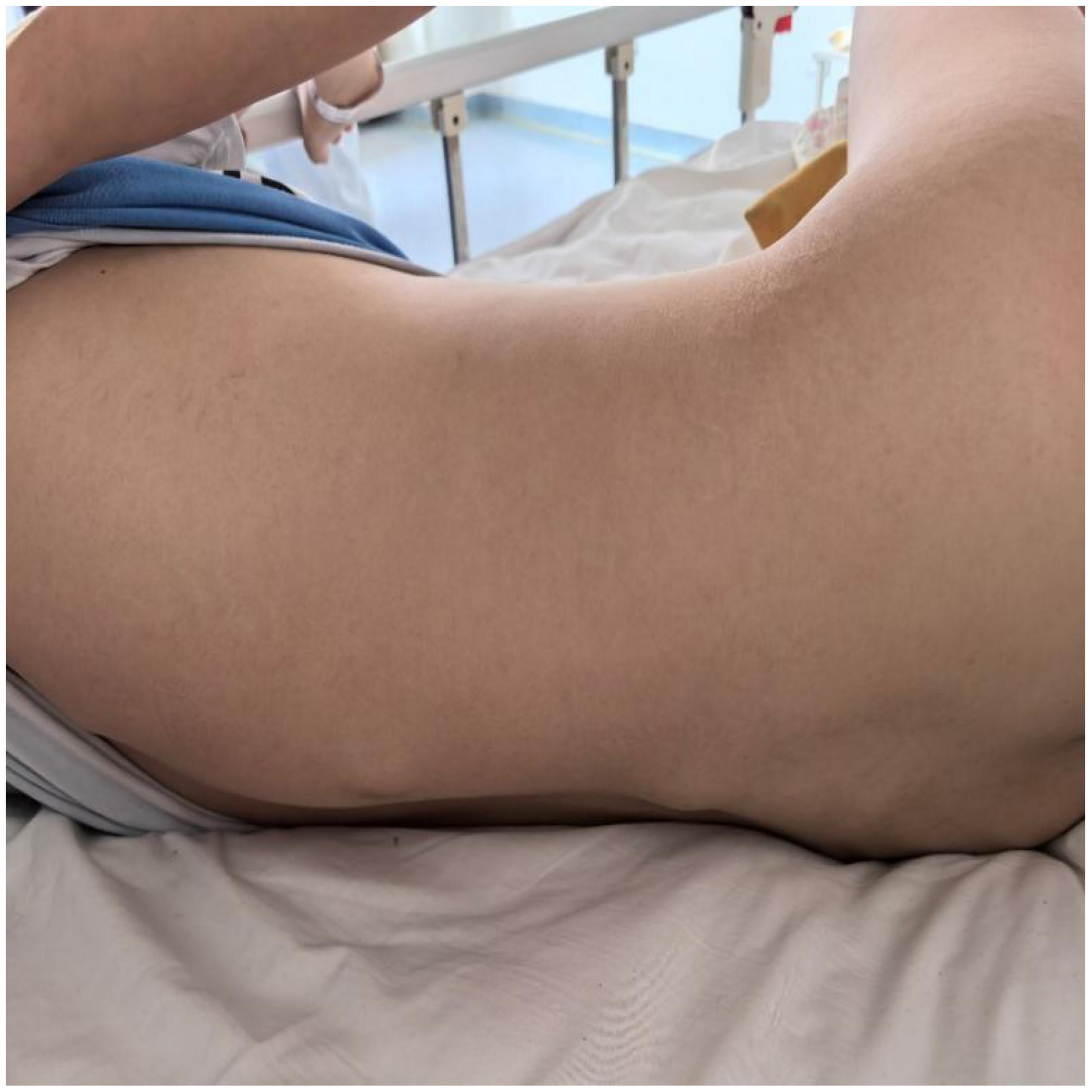
Sclerotic masses in the patient's dorsal and lumbar regions protruded from the body surface.

A comprehensive physical examination was conducted to systemically assess the burden of heterotopic ossification (HO) and its functional sequelae.

### Injured left lower limb

The left thigh presented with localized swelling, tenderness, and palpable bony crepitus. Range of motion (ROM) was severely restricted at both the hip and knee joints. Ankle and toe mobility were preserved. Distal neurovascular status, including circulation, sensation, and capillary refill, was intact.

### Contralateral right lower limb

Baseline ROM was maintained at the hip, knee, and ankle. A solid, non-tender sclerotic mass, measuring approximately 3 × 5 cm, was palpated in the right gluteal region. Pre-injury ambulation was reported, though current mobility was markedly impaired due to the left lower limb involvement.

### Spine and trunk

Prominent, protruding HO masses were evident along the para-spinal axis. A rigid, fixed mass measuring approximately 4 × 3 cm was identified in the upper-to-mid dorsal region, with another mass of about 5 × 3 cm in the lumbar region (Figure 1). These lesions resulted in severe restriction of spinal flexion, extension, and rotation. Involvement of the thoracic region contributed to visible restriction of chest wall expansion.

### Upper limbs

A firm, non-prominent sclerotic plaque is palpable around the surgical scar on the right forearm (Figure 2). Postoperative follow-up x-ray of the right forearm (Figure 3) confirmed the healed status of the prior ulnar and radial fractures, with the elastic intramedullary nails still in situ. Notably, patchy and strip-like hyperdense ossifications were observed along the nail trajectories and in the adjacent soft tissues, corresponding precisely to the palpable sclerotic plaque at the surgical site. These findings represent classic post-surgical heterotopic ossification in this patient. Shoulder and elbow ROM on the right side were moderately limited compared to the fully mobile left upper limb.

**Figure 2:**
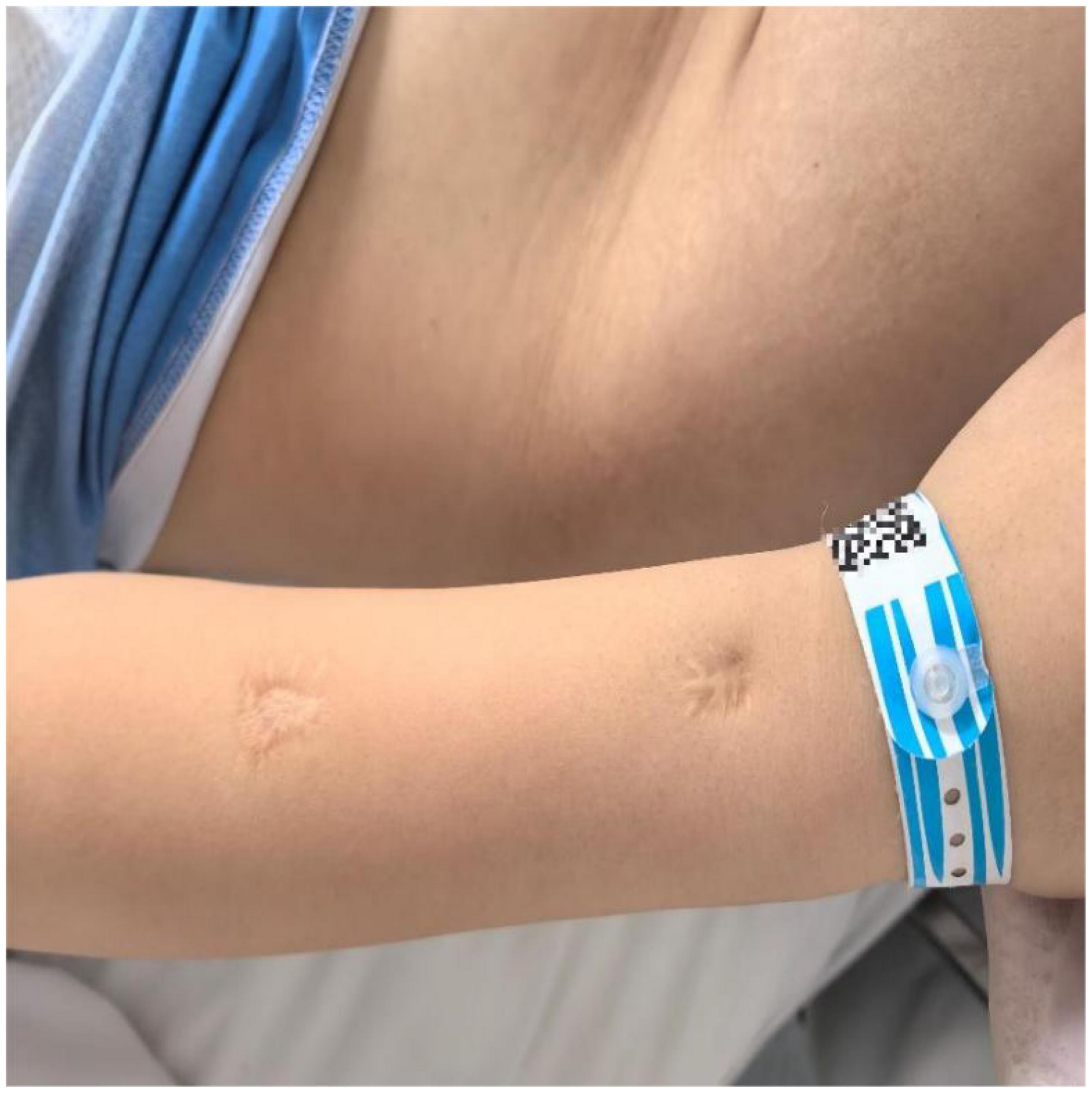
Following internal fixation surgery of the forearm at age 5, the patient developed sclerotic masses.

**Figure 3:**
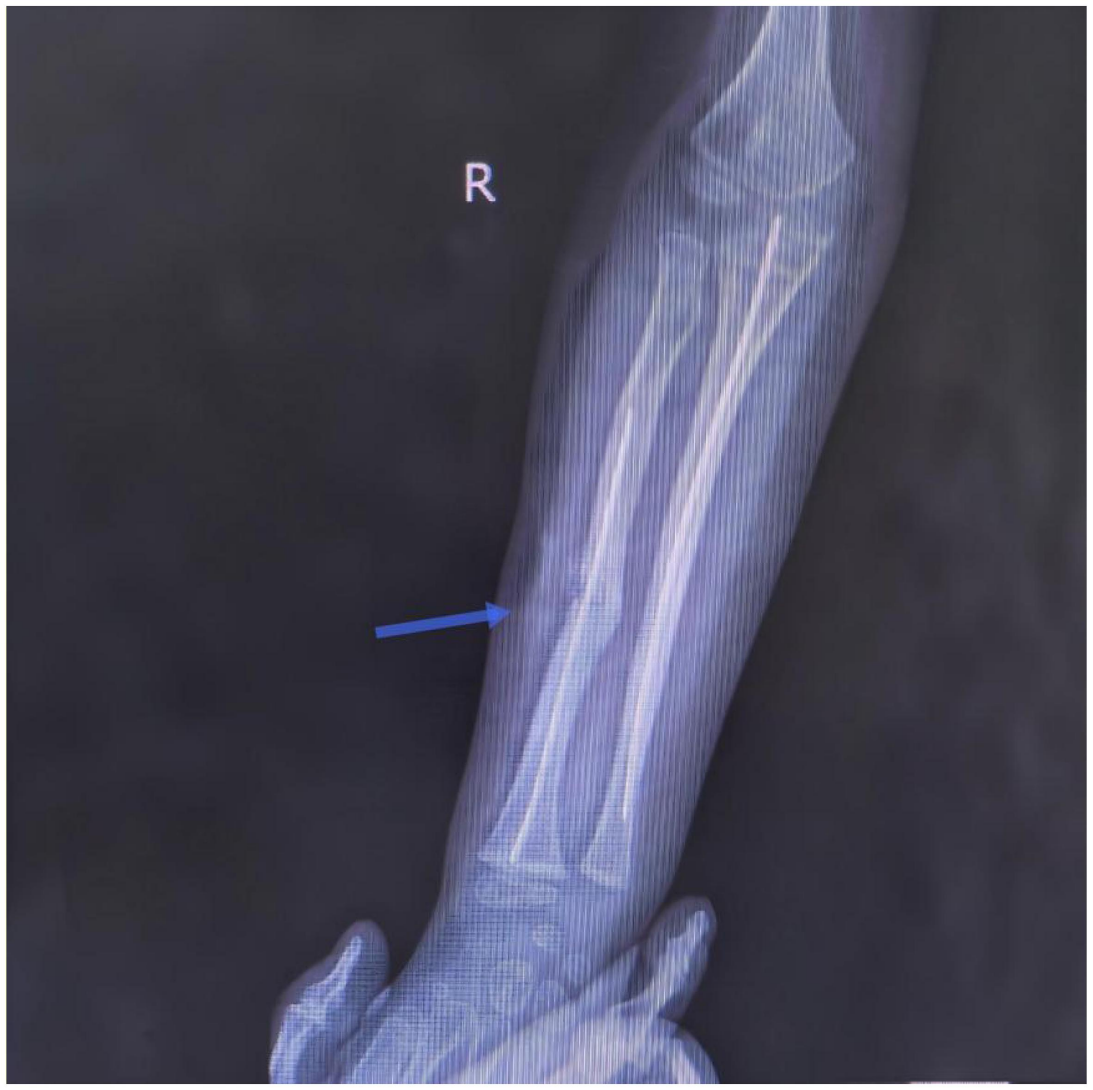
Post-operative follow-up X-ray of the right forearm (indicated by arrows).

### Additional findings

Bilateral hallux valgus deformities were present, consistent with the classic phenotype of FOP (Figure 4). No other digital anomalies or significantly enlarged lymph nodes were detected.

**Figure 4:**
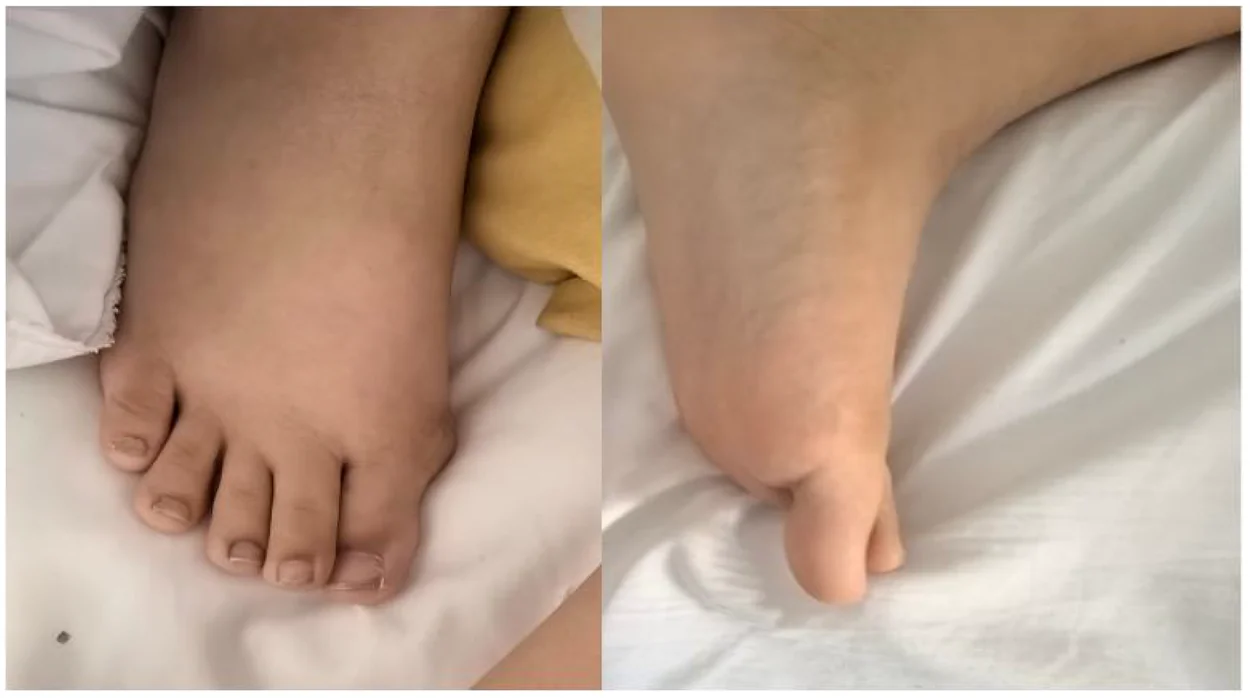
Hallux valgus imaging in the patient.

To elucidate the progression of his disease and manage the acute fracture, we reviewed imaging from previous admissions and performed new scans upon current admission, arranged in chronological order:

### Prior imaging (at age 8)

MRI of the right knee revealed a small amount of joint effusion, while anteroposterior pelvic radiography showed bilateral short and broad femoral necks with flattened and rounded femoral heads, as well as bilateral acetabular surface irregularities (Figures 5–7).

**Figure 5:**
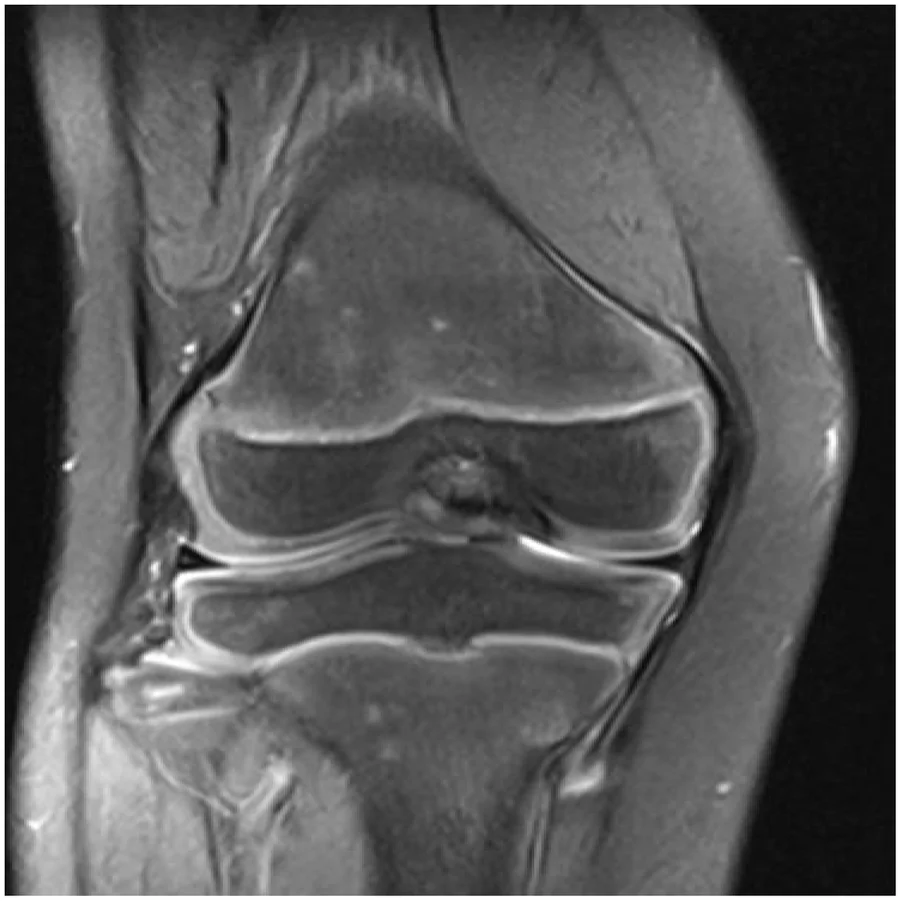
Anteroposterior MR image of the right knee joint at age 8.

**Figure 6:**
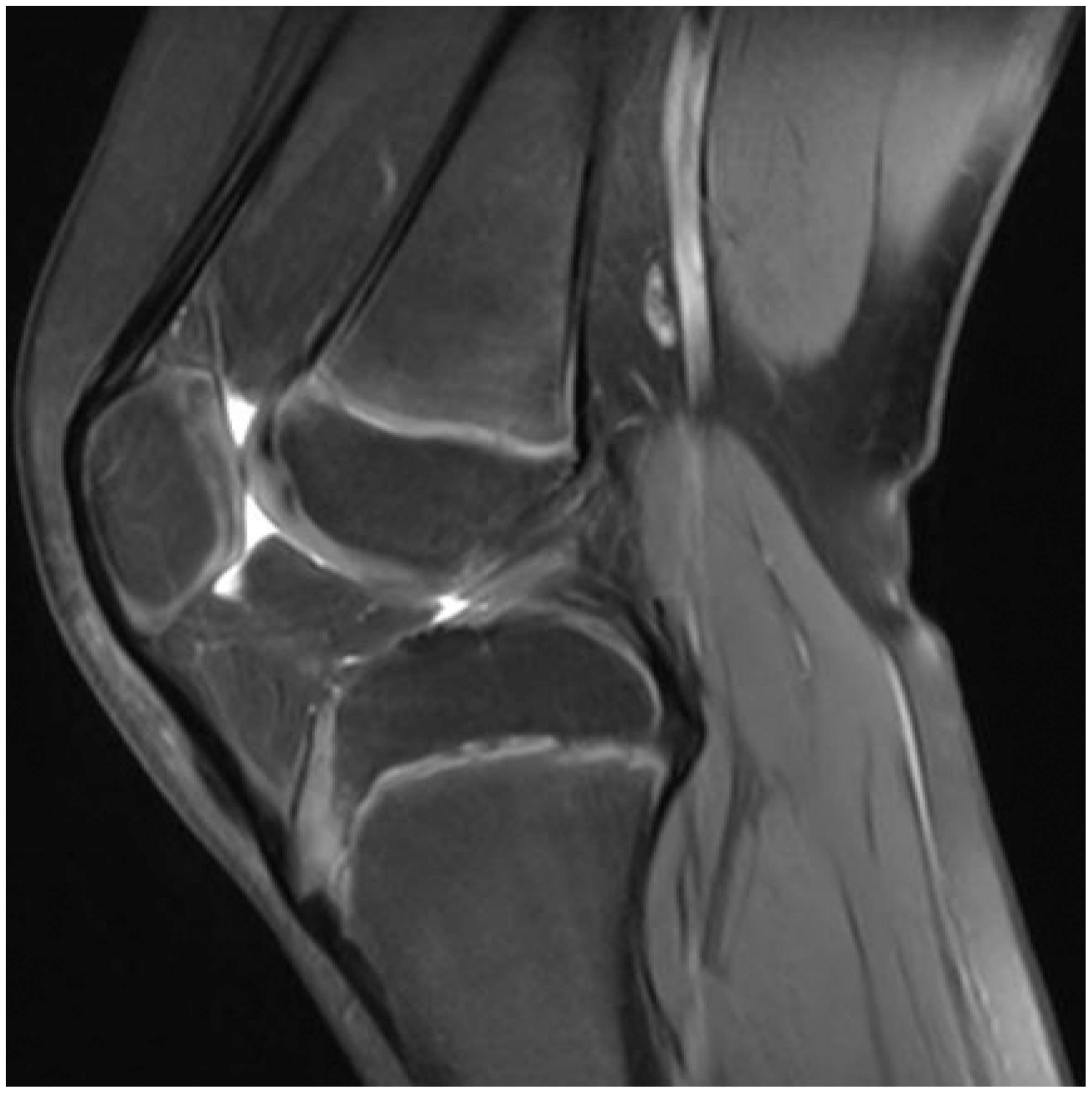
Lateral MR image of the right knee joint at age 8.

**Figure 7:**
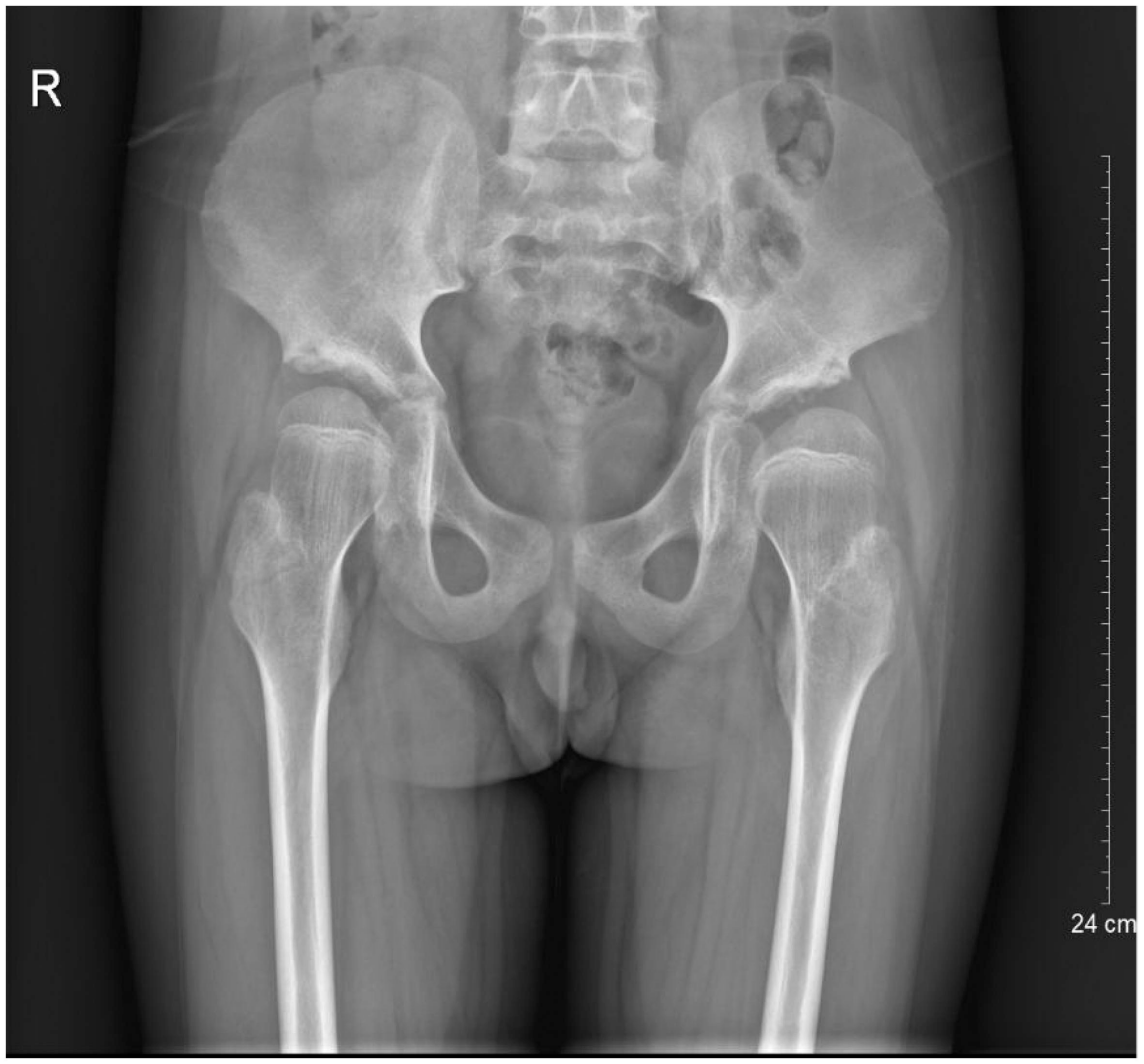
Anteroposterior pelvic radiograph at age 8.

### Imaging from one year prior to current admission (at age 11)

Anteroposterior pelvic radiography revealed irregular patchy hyperdense opacities in the left pubic/ischial rami region and ischiofemoral space. Bilateral femoral neck-shaft angles were noted to be increased (Figure 8).

**Figure 8:**
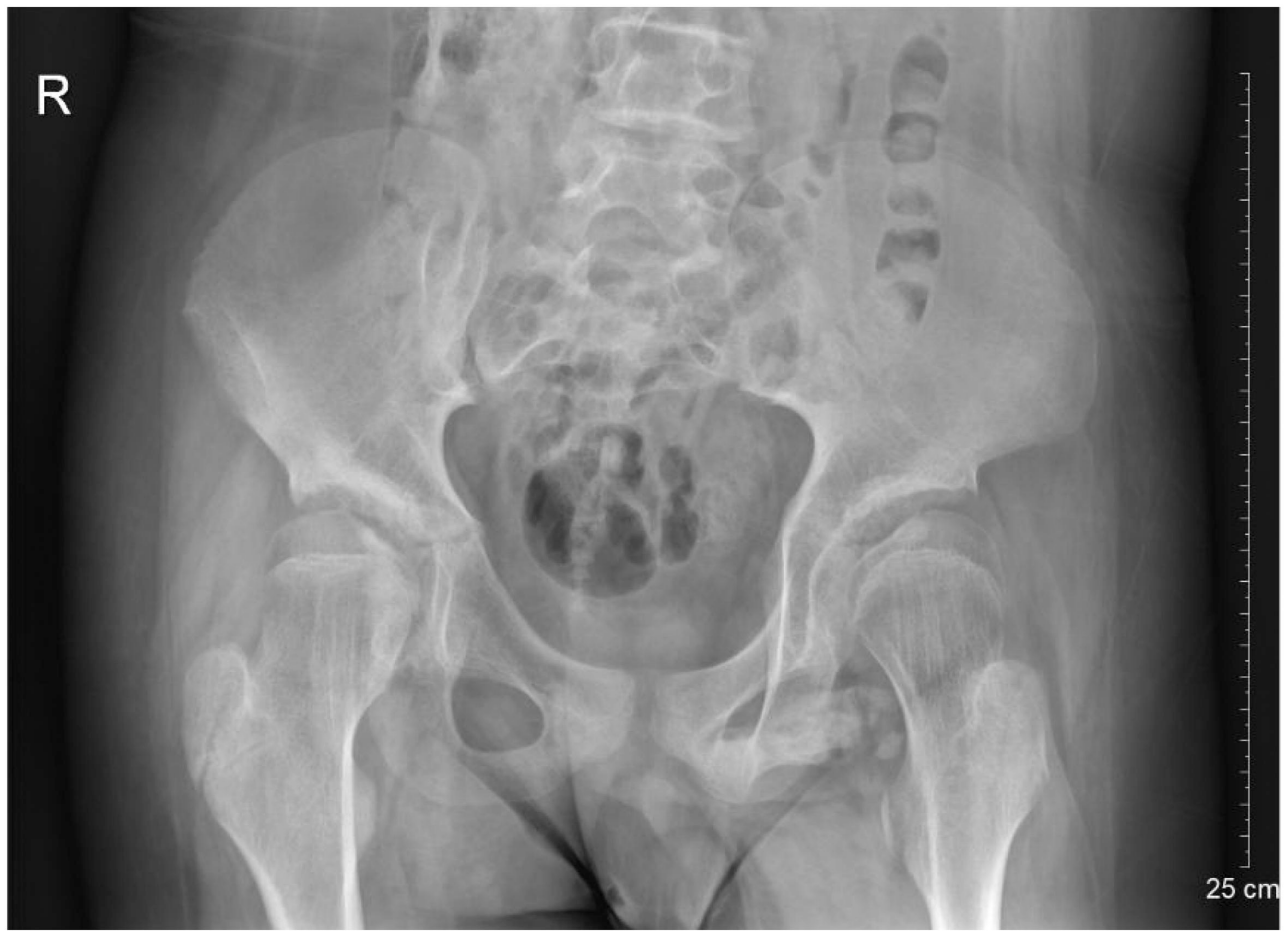
Prior pelvic radiograph (anteroposterior view) of the patient (obtained 1 year ago).

### Imaging on current admission (at age 12)

Non-contrast CT of the thigh with three-dimensional reconstruction comprehensively delineated the traumatic injury. An oblique hypodense fracture line was identified traversing the proximal-to-mid shaft of the left femur, with obvious cortical disruption. The fracture fragments demonstrated considerable separation and displacement: the distal fragment was shifted laterally and slightly posteriorly, while the proximal fragment exhibited mild anterior angulation, resulting in an overall varus deformity at the fracture site. In addition, bony protuberances originating from the left acetabulum and distal femur likely representing prior heterotopic ossification from previous injuries were observed projecting away from the articular surfaces. Despite these findings, joint congruity remained well preserved with smooth articular surfaces and maintained joint space. Prominent periarticular soft tissue swelling and a moderate hematoma were evident surrounding the fracture site, consistent with acute traumatic changes (Figures 9–11).

**Figure 9:**
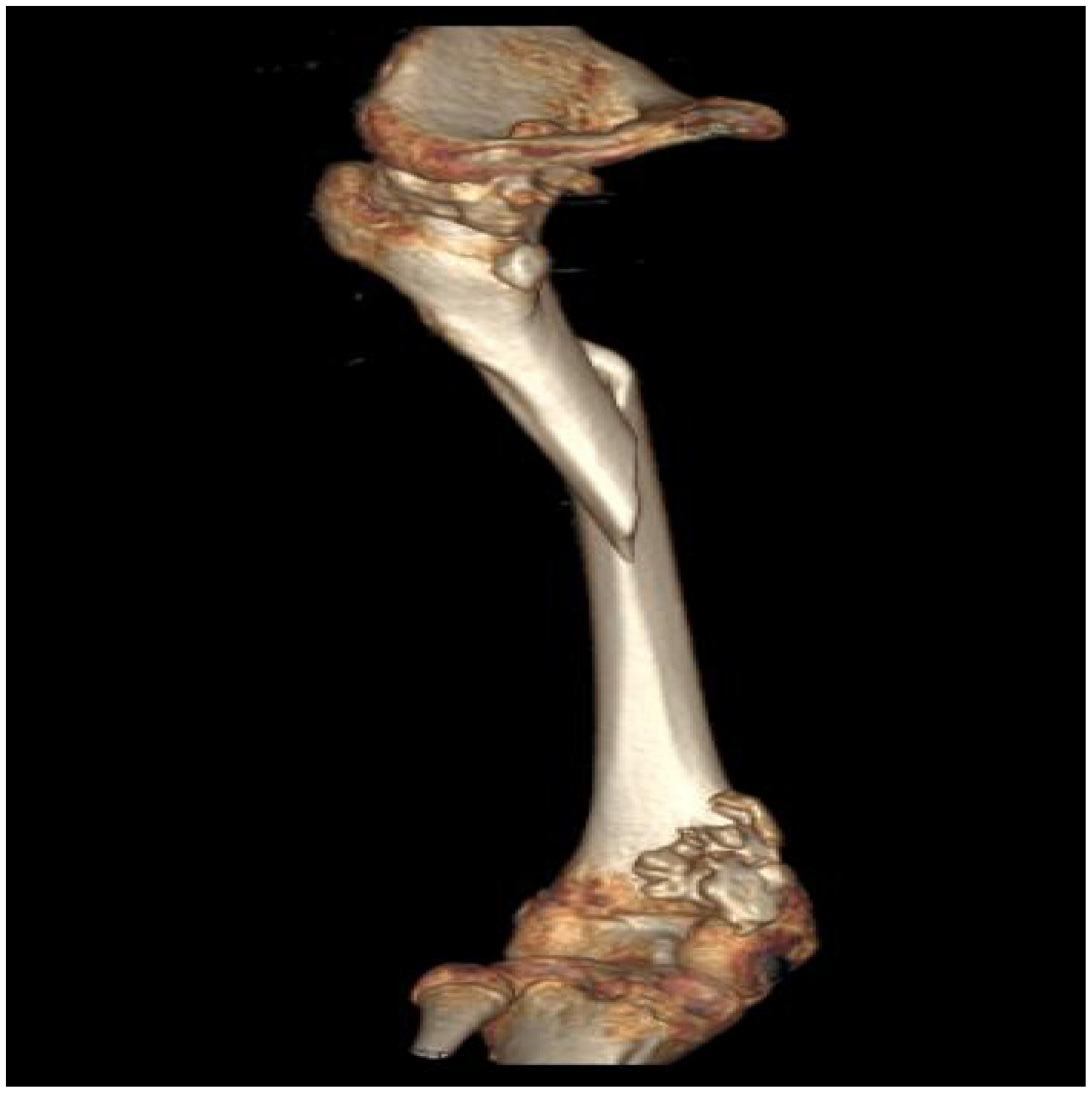
Oblique view of 3D-reconstructed CT scan of the left femur.

**Figure 10:**
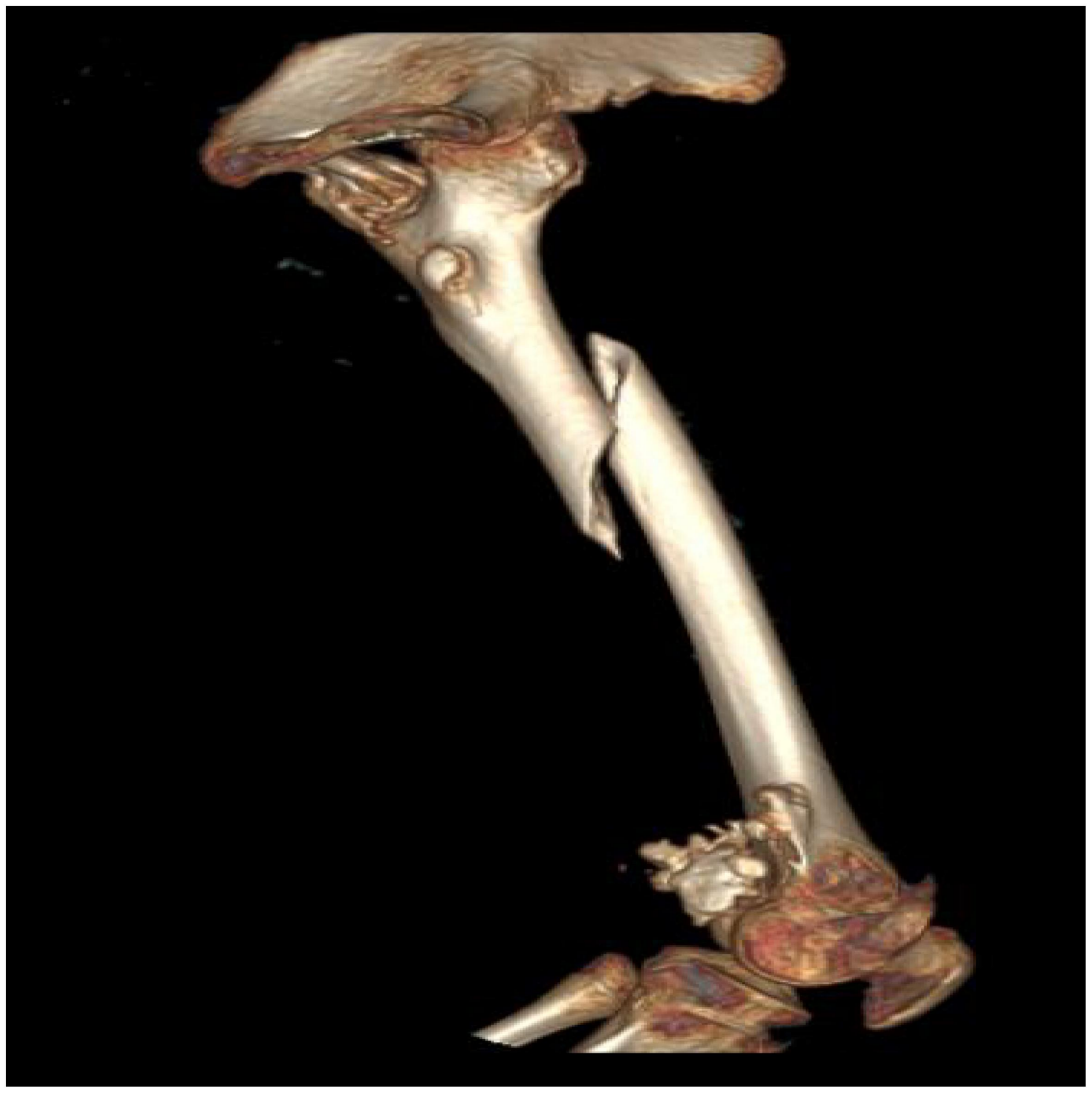
Lateral view of 3D-reconstructed CT scan of the left femur.

**Figure 11:**
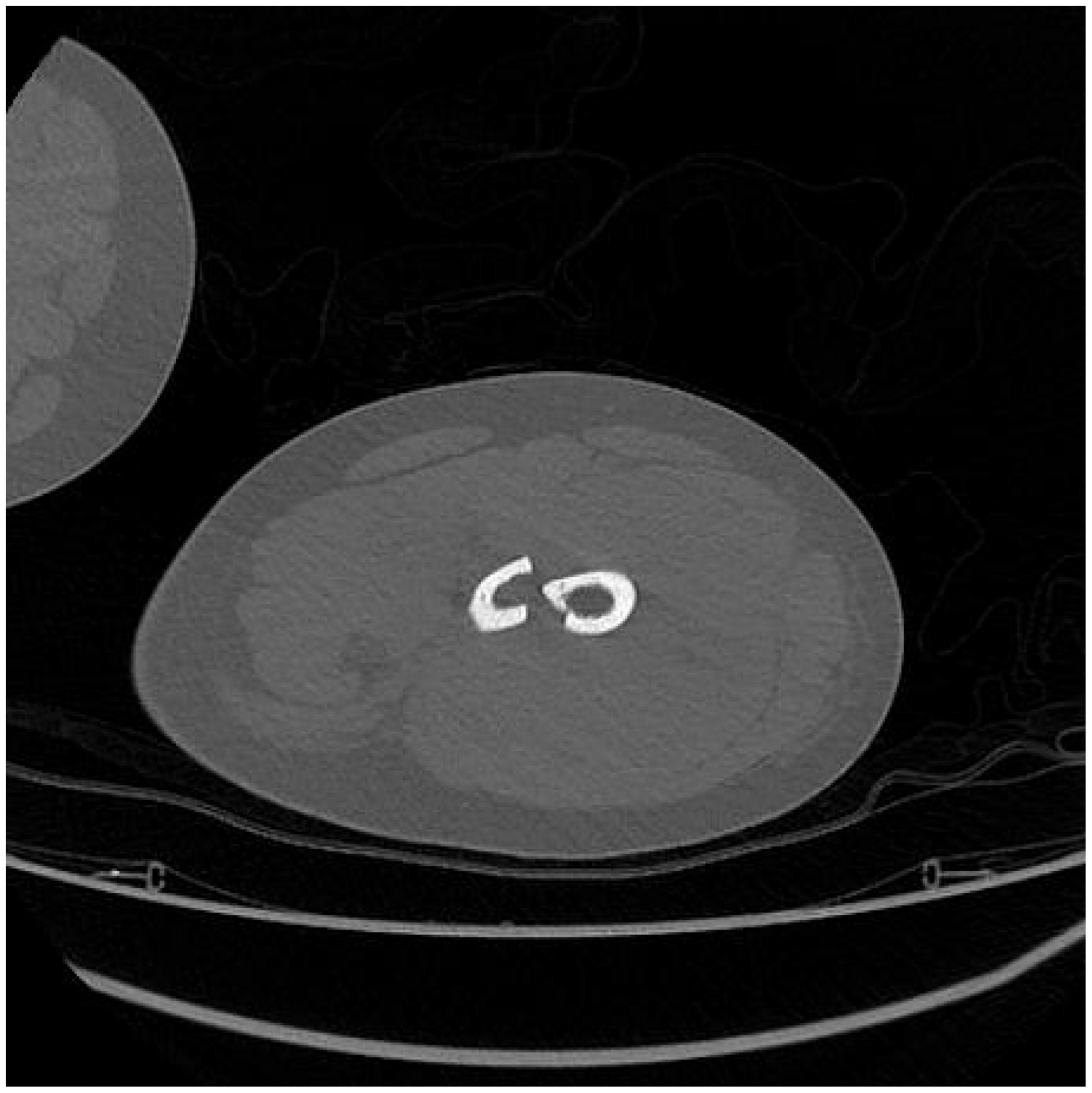
CT scan of the left femoral shaft revealed a fracture gap with separation displacement at the proximal-to-mid shaft.

Non-contrast chest CT scan revealed bilateral thoracic asymmetry with slight collapse of the left hemithorax. Irregular hyperdense opacities were observed along the right chest wall and thoracodorsal region, with partial continuity to the ribs (Figure 12).

**Figure 12:**
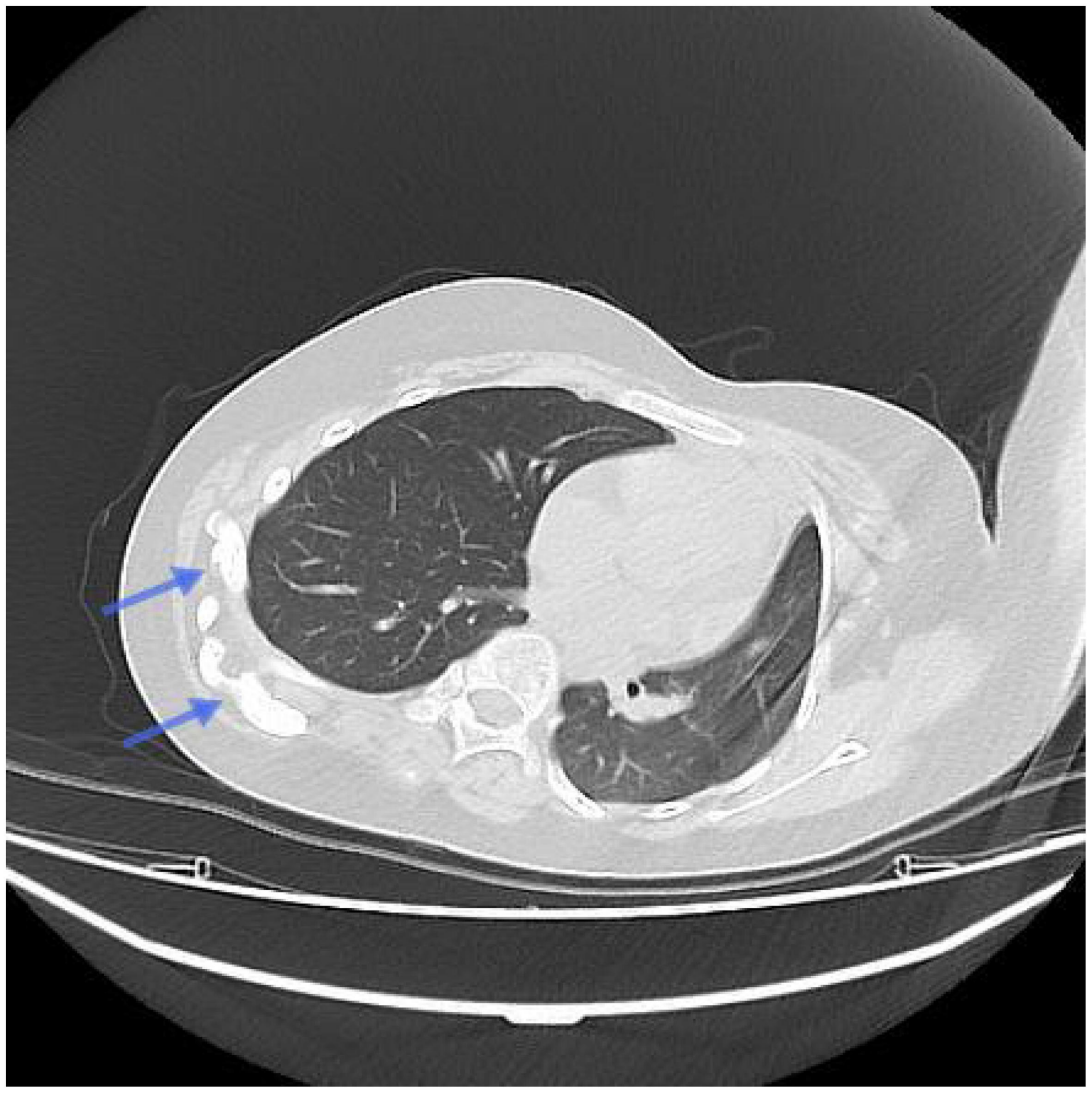
Chest CT scan (non-contrast) of the patient (indicated by arrows).

Laboratory findings revealed no significant abnormalities except for elevated inflammatory markers, plasma D-dimer, and fibrin(ogen) degradation products (FDPs)-common sequelae of acute fractures. The patient's body temperature approached 38°C during the initial two days of admission but stabilized thereafter.

Physical examinations of the patient's parents revealed no similar abnormalities, and no similar disorders are documented in the family history. Although we sought to perform confirmatory genetic analysis, these efforts were precluded by the family's socioeconomic circumstances.

Given the unique susceptibility of FOP patients, all surgical interventions except life-saving procedures should be strictly avoided. Following comprehensive evaluation and multidisciplinary consultation, closed reduction under general anesthesia with hip spica cast immobilization was selected for femoral fracture management. We educated the child and parents about FOP pathophysiology and emphasized strict prevention of trauma and invasive interventions. The child and parents consented to this treatment plan and acknowledged the educational guidance.

Although scheduled radiographic follow-up could not be performed owing to the family's socioeconomic and geographic limitations, we maintained regular clinical surveillance via telephone interviews. At 4 weeks and 12 weeks after discharge, the family reported that the patient's left thigh pain had substantially resolved, the hip spica cast remained intact and well-tolerated, and no skin breakdown or infectious complications occurred. The patient adhered strictly to non-weight-bearing status and denied any subjective sensation of fracture-site instability or abnormal mobility. No new febrile episodes or acute inflammatory flare-ups were reported during this period. These clinical parameters suggest an uneventful early recovery trajectory with conservative cast management.

## Discussion

Fibrodysplasia ossificans progressiva (FOP) is an extremely rare autosomal dominant disorder (11, 12). In 2006, Shore et al. identified the disease-causing mutation in the ACVR1/ALK2 gene, which aberrantly activates bone morphogenetic protein (BMP) signaling pathways (3). This pathological process induces progressive heterotopic ossification (HO) in muscles, tendons, and ligaments. Consequently, joint fusion and functional mobility loss occur, with most patients requiring wheelchair assistance before age 30 (13, 36, 37). The global prevalence of FOP is approximately 0.88 per million (15), though underdiagnosis and frequent misdiagnosis suggest higher actual incidence (7).

The clinical manifestations of FOP exhibit marked age-dependent progression. Characteristic great toe malformations (shortened and hallux valgus) are detectable in neonates (16, 17), a feature also present in our patient. This hallmark sign has near 100% detection rate at birth but is frequently overlooked (16). During childhood (typically 2–5 years), “flare-up inflammatory nodules” emerge as sudden, painful soft-tissue swellings resembling tumors in the neck, back, and shoulders (18). These nodules may be triggered by minor trauma (falls or injections) or occur spontaneously, often with low-grade fever, and progressively ossify over weeks. Patients commonly experience intense pain, localized swelling, erythema, and warmth during acute flare-ups, which may last for several days to weeks; as the lesion matures into bone, these acute symptoms subside, leaving a painless hard mass and progressive joint restriction. Adolescent progression accelerates heterotopic ossification, advancing axially from the trunk to limbs. Spinal ankylosis and rib fusion restrict respiratory function (19, 20). In adulthood, expanding heterotopic bone formation affects temporomandibular joints causing dysphagia and compromises thoracic mobility leading to respiratory failure—the primary cause of mortality (14, 20). In this case, recurrent traumatic events induced multifocal ossifications. The patient's lumbar/cervical spinal ankylosis, left knee joint stiffness, and restricted right shoulder mobility reflect this classic progression pattern.

Diagnosing FOP requires multidisciplinary consultations (21–23), averaging over 1.5 years with >50% misdiagnosis rate (16, 24). Key diagnostic criteria mandate concurrent progressive heterotopic ossification and congenital great toe malformations (7, 23). Imaging studies (knee/hip radiographs in this case) reveal high-density ossific foci in soft tissues and joint deformities. The histopathology of early FOP lesions demonstrates a characteristic sequence: initial intense lymphocytic infiltration, followed by fibroproliferative tissue with abundant fibroblasts and myofibroblasts, which subsequently undergoes endochondral ossification, forming mature lamellar bone with marrow elements. Critically, invasive biopsies or surgical procedures should be absolutely avoided, as even needle biopsy can provoke explosive new ossification lesions (25). If clinical and radiographic findings strongly suggest FOP, a biopsy is contraindicated and unnecessary. ACVR1 mutation detection confirms diagnosis (26, 27).

Current FOP management lacks curative treatments, requiring integrated approaches combining pharmacological intervention, injury prevention, and rehabilitative support. For high-risk populations, NSAIDs remain the gold standard for prophylaxis by inhibiting prostaglandins and directly suppressing osteoblast progression (28, 29). Glucocorticoids mitigate inflammatory responses (4), while bisphosphonates inhibit hydroxyapatite crystallization to delay ossification (30), though they carry risks of gastrointestinal/renal/cardiovascular effects, impaired bone/wound healing, electrolyte imbalances, osteonecrosis of the jaw, transient efficacy, and theoretical oncogenicity (31–37). Recent targeted drug development shows significant advances: the oral selective RARγ agonist Palovarotene blocks BMP signaling pathways and became the first drug with FDA-accepted NDA approval to potentially inhibit heterotopic ossification (38). Nevertheless, as noted in the Introduction, the EMA declined approval owing to concerns about the modest benefit-risk profile, primarily due to frequent adverse events (premature growth plate closure, epiphyseal disorders) and insufficient evidence of sustained functional improvement. This regulatory divergence underscores the need for continued research and careful patient selection. Novel agents like ACVR1 receptor inhibitors (Saracatinib) demonstrate promise in clinical trials (39).

Conservative and rehabilitative management underpins functional preservation. The cardinal principle is strict avoidance of iatrogenic harm: contraindications include intramuscular injections (including vaccinations), dental procedures, and intra-articular injections or biopsies (4, 7, 40). Patients with severe osteoporosis require fall prevention to avert pathological fractures. Despite therapeutic challenges, early recognition, prevention of misdiagnosis/mistreatment, and timely multidisciplinary collaboration (endocrinology, orthopedics, rehabilitation, genetic counseling) significantly delay complications (7, 21, 22). Scientifically guided rehabilitation protocols effectively maintain joint mobility and self-care capacity (41, 42).

### Lessons from this case

This case provides several clinically actionable insights. First, the patient's history of multiple traumas each followed by new heterotopic ossification underscores the profound vulnerability of FOP patients to even minor mechanical insults. The sequential ossification of the forearm, lumbosacral spine, and hip regions illustrates how recurrent injuries cumulatively accelerate disease burden, reinforcing the absolute necessity of trauma-avoidance education from the earliest possible age. Second, the successful closed reduction and casting of a displaced femoral shaft fracture, without surgical intervention, demonstrates that major fractures can be managed conservatively in FOP, provided that the diagnosis is known and a multidisciplinary team is engaged. This approach prevented the almost certain catastrophic HO that would have followed internal fixation. Third, the bilateral hallux valgus, present since birth but previously unrecognized, highlights the diagnostic “red flag” that clinicians must not overlook; its presence in any child with unexplained soft-tissue masses or joint contractures should prompt immediate genetic consultation and avoidance of biopsy. Finally, our experience emphasizes the critical role of family education—not only about injury prevention but also about recognizing early flare-up signs and seeking specialized care. We hope that by sharing this case, clinicians will become more vigilant in suspecting FOP, more cautious in avoiding iatrogenic harm, and more proactive in coordinating multidisciplinary care, thereby altering the natural history of this devastating condition.

## Conclusion

This case highlights that early recognition of FOP—particularly through hallmark great toe deformities—is paramount to prevent misdiagnosis and harmful procedures. Conservative fracture management, multidisciplinary collaboration, and rigorous patient/family education are cornerstones of care, and all invasive interventions must be strictly avoided to halt disease progression.

## Data Availability

The raw data supporting the conclusions of this article will be made available by the authors, without undue reservation.
